# Enhancement of Biocontrol Efficacy of Pichia kudriavzevii Induced by Ca Ascorbate against Botrytis cinerea in Cherry Tomato Fruit and the Possible Mechanisms of Action

**DOI:** 10.1128/spectrum.01507-21

**Published:** 2021-12-22

**Authors:** Keyu Sun, Ziwuzhen Wang, Xuanqing Zhang, Ze Wei, Xue Zhang, Lei Li, Yaning Fu, Jianhua Gao, Xin Zhao, Jun Guo, Junping Wang

**Affiliations:** a State Key Laboratory of Food Nutrition and Safety, Key Laboratory of Food Nutrition and Safety, Ministry of Education, Tianjin Key Laboratory of Food Nutrition and Safety, Tianjin University of Science & Technology, Tianjin, China; b Beijing Advanced Innovation Center for Food Nutrition and Human Health, Beijing Technology and Business University (BTBU), Beijing, China; c Beijing Laboratory for Food Quality and Safety, Beijing Technology and Business University, Beijing, China; d College of Life Sciences, Shanxi Agricultural University, Taigu, Shanxi, China; e Institute of Health Quarantine, Chinese Academy of Inspection and Quarantine, Beijing, China; University of Minnesota

**Keywords:** biocontrol efficacy, Ca ascorbate, cherry tomato, *Pichia kudriavzevii*

## Abstract

This study investigated the effect of Ca ascorbate on the biocontrol efficacy of Pichia kudriavzevii and the possible mechanisms. The results indicated that the biocontrol activity of *P. kudriavzevii* was significantly enhanced by 0.15 g L^−1^ of Ca ascorbate, with higher growth rates of yeast cells *in vitro* and *in vivo*. The antioxidant enzyme activity in *P. kudriavzevii*, including catalase (CAT), superoxide dismutase (SOD), and peroxidase (POD), were improved by Ca ascorbate and reached the maximum at 96 h, 96 h, and 72 h, respectively. The expression of the antioxidant enzyme-related genes *CAT1* (8.55-fold) and *SOD2* (7.26-fold) peaked at 96 h, while *PRXIID* (2.8-fold) peaked at 48 h, which were similar to the trends of enzyme activities. Compared with the control, 0.15 g L^−1^ of Ca ascorbate and CaCl_2_ increased the activity of succinate dehydrogenase in *P. kudriavzevii*, thereby enhancing the utilization of nutrients by yeast cells, and calcium ascorbate had the strongest effect. The expressions of *HXT5*, *ADH6*, *PET100p*, and *Pga62* were significantly higher in the Ca ascorbate treatment than the other groups, and the CaCl_2_ treatment was also significantly higher than the control. These results indicated that Ca ascorbate can effectively improve the energy metabolism and cell wall synthesis and slow down the senescence of yeast cells. In general, Ca ascorbate can improve the environmental adaptability of *P. kudriavzevii* and thus improve the biocontrol effect, which is associated with inducing antioxidant enzymes in yeast cells and enhancing energy metabolism and nutrient utilization efficiency to increase nutrient competition with pathogens.

**IMPORTANCE** Antagonistic yeast is a promising way to control postharvest fruit decay because of its safety and broad-spectrum resistance. However, the biocontrol efficacy of yeast is limited by environmental stress, such as oxidative stress. Therefore, the improvement of antioxidant capacity has become a research hot spot in improving the biocontrol efficacy of yeast. The induction of Ca ascorbate on the antioxidant capacity and physiological activity of yeast was studied. The results showed better induction of antioxidant enzyme and physiological activity in yeast by Ca ascorbate for better antioxidant capacity, and Ca^2+^ also played a synergistic promotion effect, which improved the biocontrol efficacy. These results provide an approach for the research and application of improving the environmental adaptability and biocontrol effectiveness of yeast.

## INTRODUCTION

Cherry tomatoes (Solanum lycopersicum var. *cerasiforme*) have higher lycopene and vitamin C and slightly higher sugar content than normal tomatoes. However, the characteristics such as rich pulp, thin skin, and soft tissue make cherry tomatoes extremely vulnerable to damage caused by mechanical impact, increasing the chance of pathogen infection (1). Gray mold decay of cherry tomato fruit, caused by Botrytis cinerea, is one of the most important postharvest diseases of cherry tomato (2). Chemical fungicides can significantly reduce postharvest losses but have adverse effects on the environment and human health, hence prompting the development of an environmentally friendly strategy has attracted wide attention (3). Since the biocontrol method was reported by Wilson (4), improving the biocontrol efficacy of original yeasts and developing novel green preservatives have become a new focus for the control of postharvest decay of fruit as an alternative to, or in combination with, chemical preservatives (5).

Competition for nutrients and space is an important mechanism of antagonistic yeast to prevent and control postharvest diseases (6). Fungal infection causes excessive reactive oxygen species (ROS) production in fruit and leads to oxidative stress to the fungus, which can rapidly oxidize and damage lipids in cellular membranes, as well as proteins and other cellular components (7), leading to cellular dysfunction and ultimately to fungal cell senescence (8) and death (9). Reducing ROS stress of yeast or the enhancing antioxidant activity of antagonistic yeast has become a research hot spot in recent years. The induced treatment of yeast by adding glycine betaine (10), glutathione (11), and chitosan (12) into the culture medium has been reported and could induce an increase in the antioxidant capacity of yeast cells. Under oxidative stress conditions, the direct effect of vitamin C on Pichia caribbica had been shown with improvement of biocontrol activity in the similarly induced treatment mentioned above, which is related to the enhancement of yeast antioxidative enzymes (13). Compared with vitamin C, Ca ascorbate has more stable properties, and Ca^2+^ could enhance the antioxidant capacity of Ca ascorbate (14). Ca ascorbate, a widely used antioxidant, plays a crucial role in reducing browning, delaying senescence, and prolonging the shelf life of fresh-cut rose apple (15). Ca^2+^ takes part in regulating the physiological activities of cells. A sufficient concentration of cellular calcium is necessary for mitotic cells to go through the G_1_ and G_2_/M phases; indeed, in Saccharomyces cerevisiae cells, endogenous Ca^2+^ plays a role in the mitotic cycle and the mating process (16). Previous studies focused on the direct inhibitory effect on the pathogen of exogenous Ca^2+^, which can inhibit the spore germination and germ tube elongation of pathogens such as Alternaria alternata (17) and Penicillium expansum (18) and combined use with antagonistic yeast to control fruit decay (19, 20). However, the effects of antioxidant Ca ascorbate and exogenous Ca^2+^-induced treatment on the proliferation, basic physiological characteristics, and biocontrol effect of yeast have not been well studied.

This study investigated the effect of Ca ascorbate on improving the biocontrol efficacy of Pichia kudriavzevii in inhibiting *Botrytis cinerea* in tomato and a possible underlying mechanism. The specific objectives were to assess (i) the effect of Ca ascorbate on the activity of *P. kudriavzevii* in controlling gray mold decay of cherry tomato fruit, (ii) the effect of Ca ascorbate induction on the activity and gene expression of antioxidant enzymes (including catalase [CAT], superoxide dismutase [SOD], and peroxidase [POD]) in *P. kudriavzevii*, and (iii) the effects of Ca ascorbate treatment on proliferation capacity (population dynamics and expression of the related gene *Pga62*) and nutrient utilization (metabolic activity and expression of the related genes *ADH6*, *HXT5*, and *PET100p*) of *P. kudriavzevii*.

## RESULTS

### Ca ascorbate improved the biocontrol efficacy of *P. kudriavzevii* against postharvest gray mold decay of cherry tomato fruit.

**FIG 1:**
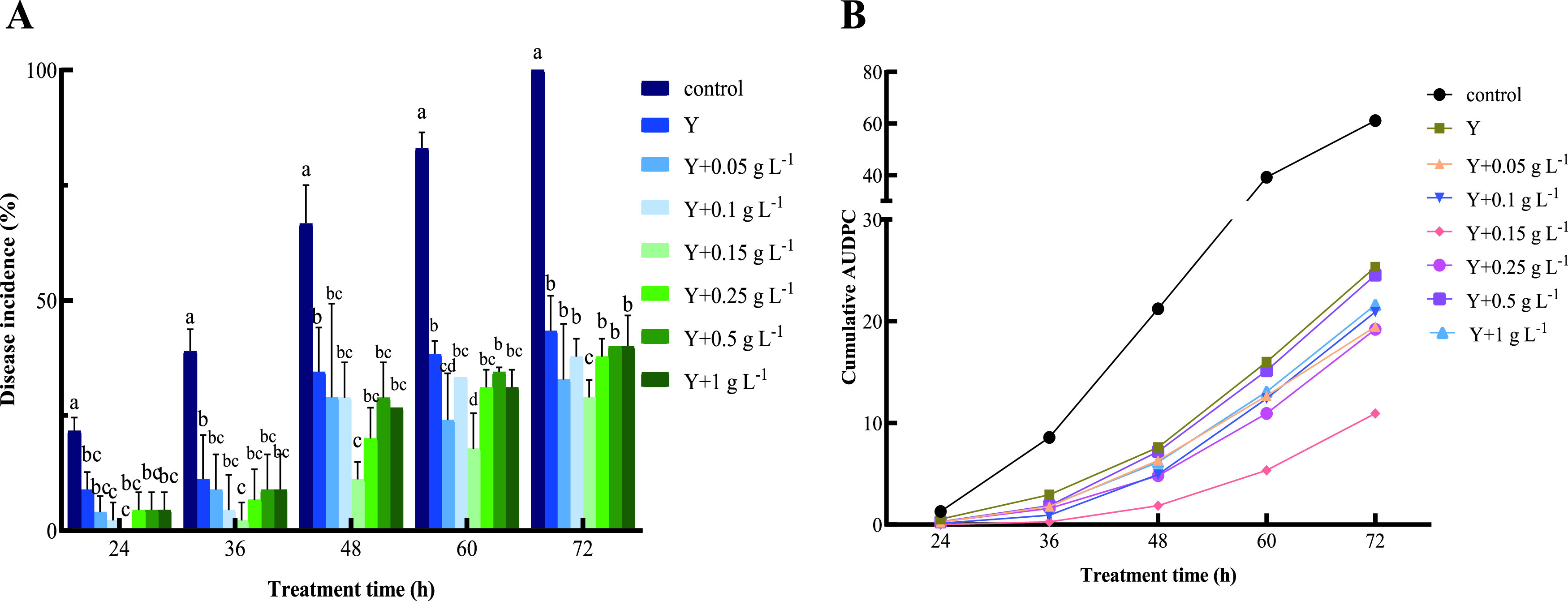
Ca ascorbate enhanced the biocontrol effect of *P. kudriavzevii* against postharvest gray mold decay of cherry tomato fruit. (A and B) Disease incidence (A) and the area under the disease progress curve (AUDPC) (B) were measured and calculated after *B. cinerea* inoculation and incubation at 28°C, RH 95%. Control: sterile water; Y: *P. kudriavzevii*; Y + 0.05 g L^−1^: NYDB was supplemented with 0.05 g L^−1^ Ca ascorbate; Y + 0.1 g L^−1^: NYDB was supplemented with 0.1 g L^−1^ Ca ascorbate; Y + 0.15 g L^−1^: NYDB was supplemented with 0.15 g L^−1^ Ca ascorbate; Y + 0.25 g L^−1^: NYDB was supplemented with 0.25 g L^−1^ Ca ascorbate; Y + 0.5 g L^−1^: NYDB was supplemented with 0.5 g L^−1^ Ca ascorbate; Y + 1 g L^−1^: NYDB was supplemented with 1 g L^−1^ Ca ascorbate. Bars represent the standard errors based on three replications. Different letters indicate significant differences (*P < *0.05) determined by the Duncan’s multiple-range test.

As shown in Fig. 1A, *P. kudriavzevii* effectively controlled postharvest gray mold decay of tomato caused by *Botrytis cinerea*. However, yeasts induced by 0.15 g L^−1^ Ca ascorbate exhibited a better biocontrol efficacy than that of other concentrations of Ca ascorbate-induced yeasts and non-Ca ascorbate-induced yeasts. At 24, 36, 48, 60, and 72 h, the disease incidence of the non-Ca ascorbate-induced yeast treatment group and the Ca ascorbate-induced yeast treatment groups were significantly lower than that of the control, and the 0.15 g L^−1^ Ca ascorbate-induced yeast group was the lowest. Disease incidences in cherry tomato treated with noninduced yeast and 0.15 g L^−1^ Ca ascorbate-induced yeast were 43.33% and 28.9%, respectively, whereas disease incidence in the control fruits (inoculated with water followed by the pathogen) reached 100% at 72 h. The area under the disease progress curve (AUDPC) is a quantitative tool for measuring harvest losses due to pathogen attack. Fig. 1B shows that the AUDPC was significantly reduced in yeast treatment groups compared with that of the control, and the 0.15 g L^−1^ Ca ascorbate-induced yeast treatment group was the lowest. The trend of AUDPC of fruits was consistent with the trend of disease incidence. Thus, *P. kudriavzevii* induced by 0.15 g L^−1^ Ca ascorbate was used for further experiments in this study.

### Ca ascorbate, CaCl_2_, and vitamin C accelerated population dynamics and cell growth rates of *P. kudriavzevii*.

**FIG 2:**
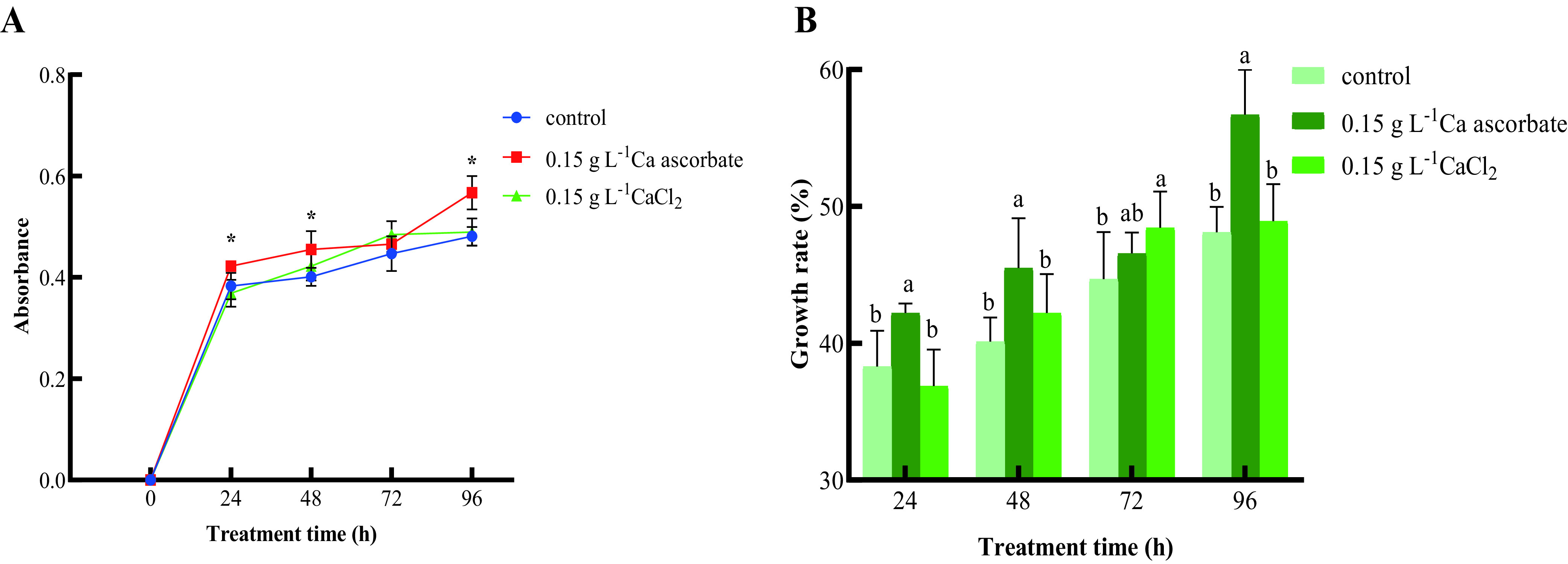
(A and B) The population dynamics (A) and cell growth rate (B) of *P. kudriavzevii in vitro*. Control: *P. kudriavzevii* cultured in NYDB; 0.15 g L^−1^ Ca ascorbate: *P. kudriavzevii* cultured in NYDB supplemented with 0.15 g L^−1^ of Ca ascorbate; 0.15 g L^−1^ CaCl_2_: *P. kudriavzevii* cultured in NYDB supplemented with 0.15 g L^−1^ of CaCl_2_. Bars represent the standard errors based on three replications. Asterisks (*) and different letters indicate significant differences (*P < *0.05) compared to the control.

**(i) *In vitro* test.** Fig. 2A shows that the population dynamics of *P. kudriavzevii* induced by Ca ascorbate and CaCl_2_ was increased in nutrient yeast dextrose broth medium (NYDB). The number of cells in the 0.15 g L^−1^ Ca ascorbate treatment group increased markedly at 24, 48, and 96 h, reaching the maximum at 96 h. The amount of *P. kudriavzevii* in the CaCl_2_ treatment group was higher than that in the control and reached the maximum at 72 h.

Figure 2B shows that the yeast growth rate in all the treatments increased, reaching the highest value at 96 h. At 72 h, the growth rate of the CaCl_2_ treatment group was the highest, at 48.44%. The yeast growth rate was highest in the Ca ascorbate treatment group at all other time points, reaching the maximum at 96 h (56.72%).

**FIG 3:**
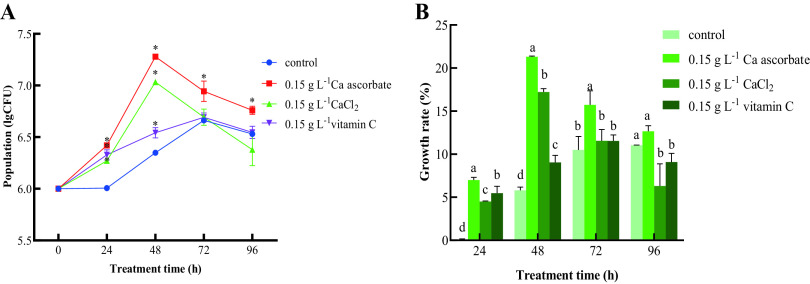
(A and B) The population dynamics (A) and cell growth rate (B) of *P. kudriavzevii in vivo*. Control: *P. kudriavzevii* cultured in NYDB; 0.15 g L^−1^ Ca ascorbate: *P. kudriavzevii* cultured in NYDB supplemented with 0.15 g L^−1^ of Ca ascorbate; 0.15 g L^−1^ CaCl_2_: *P. kudriavzevii* cultured in NYDB supplemented with 0.15 g L^−1^ of CaCl_2_; 0.15 g L^−1^ vitamin C: *P. kudriavzevii* cultured in NYDB supplemented with 0.15 g L^−1^ of vitamin C. Bars represent the standard errors based on three replications. Asterisks (*) and different letters indicate significant differences (*P < *0.05) compared to the control.

**(ii) *In vivo* test.** As shown in Fig. 3A, the population of *P. kudriavzevii* treated with Ca ascorbate and CaCl_2_ on the surface of cherry tomato fruit increased faster than that of the control. The population of *P. kudriavzevii* induced by 0.15 g L^−1^ Ca ascorbate and CaCl_2_ multiplied rapidly from 0 to 48 h (*P* < 0.05), reaching the maximum (7.45 log_10_ CFU circle^−1^ and 7.03 log_10_ CFU circle^−1^), and then decreased. In general, the yeast population in the 0.15 g L^−1^ Ca ascorbate treatment was the largest during the whole experiment. Treatment with 0.15 g L^−1^ vitamin C also increased the number of *P. kudriavzevii*, reaching the maximum at 72 h (6.55 log_10_ CFU circle^−1^), but the effect was not as good as Ca ascorbate; meanwhile, the control also reached a maximum (6.53 log_10_ CFU circle^−1^).

Figure 3B showed that the growth rate of yeasts induced by all treatments increased first and then decreased, while the growth rate of the control group continued to increase. The growth rates of the Ca ascorbate and the CaCl_2_ treatment groups were highest at 48 h (reaching 21.32% and 17.21%, respectively), while the growth rate in the vitamin C treatment group reached a peak at 72 h (up to 11.53%). In general, *P. kudriavzevii* induced by Ca ascorbate showed the highest growth rate on the fruit surface throughout the experiment; the CaCl_2_ and vitamin C treatment groups also had a higher colonization rate than the control in the initial stage.

### Ca ascorbate enhanced antioxidant enzyme activities of *P. kudriavzevii*.

**FIG 4:**
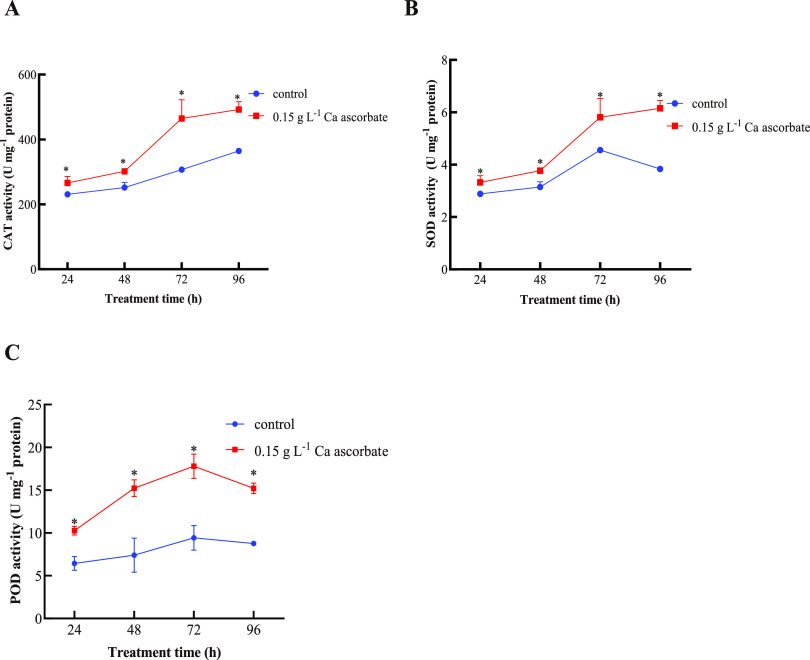
(A to C) Determination of antioxidant enzyme activity of CAT (A), SOD (B), and POD (C). Control: *P. kudriavzevii* cultured in NYDB; 0.15 g L^−1^ Ca ascorbate: *P. kudriavzevii* cultured in NYDB supplemented with 0.15 g L^−1^ of Ca ascorbate. Each value is the mean of three replications. Bars represent the standard error of the mean. Asterisks (*) indicate significant differences (*P < *0.05) compared to the control.

The results in Fig. 4 show that Ca ascorbate significantly enhanced the CAT, SOD, and POD activities in *P. kudriavzevii* compared with the control. The CAT activity in *P. kudriavzevii* gradually increased in both groups and reached the maximum at 96 h. The CAT activity in *P. kudriavzevii* induced by Ca ascorbate was higher throughout the experimental period (Fig. 4A) compared with the control. Ca ascorbate treatment increased the SOD activity in *P. kudriavzevii* at all the tested time points, reaching the maximum at 96 h (Fig. 4B). POD activity in both groups increased gradually to the maximum at 72 h and then decreased. The Ca ascorbate treatment group always maintained a higher level of activity (Fig. 4C).

### Relative expression levels of antioxidant enzyme genes.

**FIG 5:**
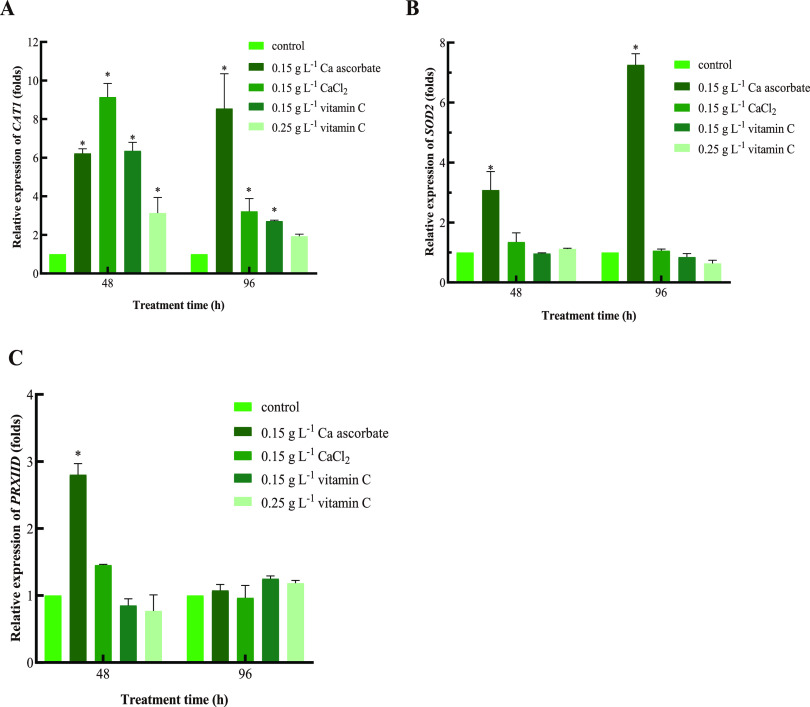
(A to C) The relative expression of *CAT1* (A), *SOD2* (B), and *PRXIID* (C) in *P. kudriavzevii*. β-actin was used as an endogenous reference gene. Control: *P. kudriavzevii* cultured in NYDB; 0.15 g L^−1^ Ca ascorbate: *P. kudriavzevii* cultured in NYDB supplemented with 0.15 g L^−1^ of Ca ascorbate; 0.15 g L^−1^ CaCl_2_: *P. kudriavzevii* cultured in NYDB supplemented with 0.15 g L^−1^ of CaCl_2_; 0.15 g L^−1^ vitamin C: *P. kudriavzevii* cultured in NYDB supplemented with 0.15 g L^−1^ of vitamin C; 0.25 g L^−1^ vitamin C: *P. kudriavzevii* cultured in NYDB supplemented with 0.25 g L^−1^ of vitamin C. Values were normalized to the control. Bars represent the standard errors based on three replications. The asterisks (*) indicate relative transcript levels that were significantly (*P < *0.05) higher (increased by more than 2.0-fold) in relation to the control.

The gene expression levels of CAT1, SOD2 and PRXIID of yeasts in different treatments were shown in Fig. 5. Compared with the control, in the 0.15 g L^−1^ Ca ascorbate treatment group, the transcription of *CAT1* and *SOD2* was significantly upregulated throughout the experimental period and reached the peak (8.6- and 7.3-fold) at 96 h. In the 0.15 g L^−1^ CaCl_2_ treatment group, the relative transcription level of *CAT1* was upregulated and reached the peak (9.14-fold) at 48 h compared with the control. Treatment with 0.15 g L^−1^ and 0.25 g L^−1^ vitamin C also significantly increased the relative expression of the *CAT1* gene at 48 h but was lower than that of the Ca ascorbate and CaCl_2_ treatment groups. The treatment with 0.15 g L^−1^ Ca ascorbate resulted in higher relative expression of *PRXIID* (2.8-fold) at 48 h compared with the control and the other treatment groups. The relative expression of *SOD2* and *PRXIID* did not change significantly in the CaCl_2_ and vitamin C treatment groups compared with the control.

### Ca ascorbate increased metabolic activity of *P. kudriavzevii*.

**FIG 6:**
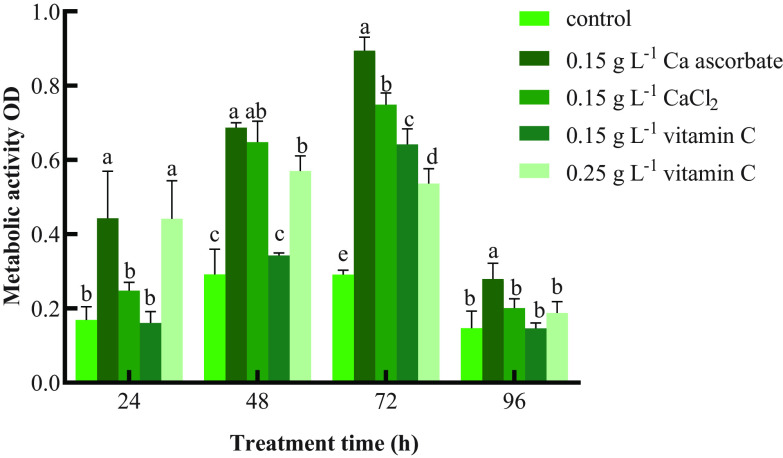
Metabolic activity of *P. kudriavzevii*. Control: *P. kudriavzevii* cultured in NYDB; 0.15 g L^−1^ Ca ascorbate: *P. kudriavzevii* cultured in NYDB supplemented with 0.15 g L^−1^ of Ca ascorbate; 0.15 g L^−1^ CaCl_2_: *P. kudriavzevii* cultured in NYDB supplemented with 0.15 g L^−1^ of CaCl_2_; 0.15 g L^−1^ vitamin C: *P. kudriavzevii* cultured in NYDB supplemented with 0.15 g L^−1^ of vitamin C; 0.25 g L^−1^ vitamin C: *P. kudriavzevii* cultured in NYDB supplemented with 0.25 g L^−1^ of vitamin C. Each value is the mean of three replications. Bars represent the standard error of the mean. Data in columns with different letters are significantly different according to Duncan’s multiple-range test at *P < *0.05.

Figure 6 shows that the metabolic activity of all treatment groups gradually increased and peaked at 72 h, with all treatments showing significantly higher activity than the control. Throughout the experimental period, the metabolic activity was highest in the 0.15 g L^−1^ Ca ascorbate treatment group. Compared with the control, the CaCl_2_ treatment obviously increased the metabolic activity of *P. kudriavzevii* at 48 and 72 h. Treatment with 0.25 g L^−1^ vitamin C also obviously increased the metabolic activity of *P. kudriavzevii* except for at 96 h, while the metabolic activity of yeast was significantly increased only at 72 h after 0.15 g L^−1^ vitamin C induction compared with the control. At 72 h, there were significant differences in metabolic capacity among all the treatment groups. The sequence of treatments regarding the improvement of yeast metabolic capacity was Ca ascorbate > CaCl_2_ > vitamin C.

### Ca ascorbate increased the relative expression levels of genes involved in cell growth and basic activity.

**FIG 7:**
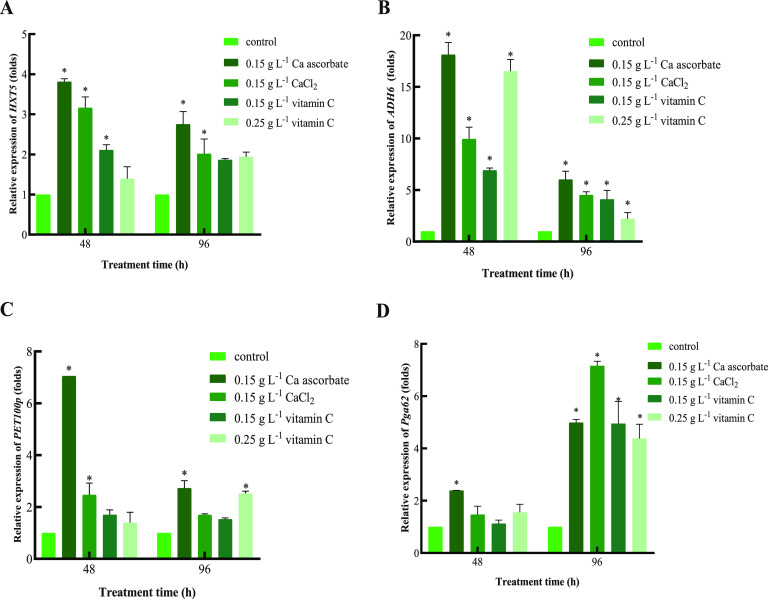
(A to D) The relative expression of *HXT5* (A), *ADH6* (B), *PET100p* (C), and *Pga62* (D) in *P. kudriavzevii*. β-actin was used as an endogenous reference gene. Control: *P. kudriavzevii* cultured in NYDB; 0.15 g L^−1^ Ca ascorbate: *P. kudriavzevii* supplemented with NYDB amended with 0.15 g L^−1^ of Ca ascorbate; 0.15 g L^−1^ CaCl_2_: *P. kudriavzevii* supplemented with NYDB amended with 0.15 g L^−1^ of CaCl_2_; 0.15 g L^−1^ vitamin C: *P. kudriavzevii* cultured in NYDB supplemented with 0.15 g L^−1^ of vitamin C; 0.25 g L^−1^ vitamin C: *P. kudriavzevii* cultured in NYDB supplemented with 0.25 g L^−1^ of vitamin C. Values were normalized to the control. Bars represent the standard errors based on three replications. The asterisks (*) indicate relative transcript levels that were significantly (*P < *0.05) higher (increased by more than 2.0-fold) in relation to the control.

As can be seen from Fig. 7, compared with the control, in the 0.15 g L^−1^ Ca ascorbate treatment, the transcription of *HXT5*, *ADH6*, *PET100p*, and *Pga62* was significantly upregulated throughout the experimental period, and the 0.15 g L^−1^ CaCl_2_ treatment group also showed a similar trend. Vitamin C also increased the expression of these genes, but not as much as Ca ascorbate and CaCl_2_. The expression levels of *HXT5*, *ADH6*, and *PET100p* in yeast treated with 0.15 g L^−1^ Ca ascorbate were highest at 48 h (3.81-, 18.12-, and 7.05-fold, respectively), and in the 0.15 g L^−1^ CaCl_2_ group the expression levels reached their peak at 48 h (3.16-, 9.95-, and 2.47-fold, respectively). The *HXT5*, *ADH6* and *PET100p* gene expression levels were higher in the Ca ascorbate treatment than the CaCl2 treatment. In the 0.15 g L^−1^ vitamin C treatment group, the relative transcription levels of *HXT5* and *ADH6* were significantly increased at 48 h (2.11- and 6.91-fold, respectively). In the 0.25 g L^−1^ vitamin C treatment group, the relative transcription of *ADH6* was significantly upregulated, reaching the peak at 48 h (16.51-fold), whereas the relative transcription levels of *PET100p* were increased at 96 h (2.52-fold). The 0.15 g L^−1^ Ca ascorbate treatment also induced higher relative expression of *Pga62*, reaching the peak at 96 h (4.99-fold increase); at that time, the gene expression was highest in the CaCl_2_-treated group (6.96-fold increase). The relative expression of *Pga62* was also upregulated at 96 h (4.95-fold and 4.38-fold, respectively) in both the 0.15 g L^−1^ vitamin C and 0.25 g L^−1^ vitamin C treatment groups, but not as much as Ca ascorbate and CaCl_2_.

## DISCUSSION

In recent years, yeast biological control capacity has been explored to protect postharvest fruit from pathogen infection and to meet people’s quest for healthy food (21). However, the biocontrol efficacy of yeast is not as reliable as the use of chemical fungicides, prompting researchers to consider treating yeasts with chemical agents to enhance the yeasts’ effects (22).

The research on methods of improving the biocontrol efficacy of yeast has become one of the hot topics in the field of postharvest diseases control with antagonistic yeasts. In recent years, it has been reported that antioxidants can enhance the biological control effect of antagonistic yeast. Zhang et al. (23) reported that culturing Cryptococcus laurentii with exogenous glutathione reduced the incidence of disease caused by *Penicillium* in pears. Chitin induction can improve the biological control ability of Rhodotorula mucilaginosa against strawberry postharvest disease (24). In our study, results indicated that 0.15 g L^−1^ of Ca ascorbate significantly enhanced the biocontrol efficacy of *P. kudriavzevii* against gray mold in cherry tomato fruits.

The accumulation of exogenous reactive oxygen species (ROS) and internal ROS is one of the main factors affecting yeast activity and biocontrol effects (6, 25). Biological stress promotes the generation of ROS in fruit tissues and causes metabolic disorders in the body, leading to a decline in cell activity (26). Excessive ROS in cells can cause oxidative damage to proteins, lipids, and nucleic acids, leading to structural and functional damage (27). Therefore, improving the tolerance of antagonistic yeast cells to oxidative stress has become one of the key issues to improve their biocontrol effectiveness.

In order to reduce excessive ROS accumulation, yeast has evolved an antioxidant defense system that contains various enzymes and nonenzymatic components. Antioxidant enzymes that can directly react with and eliminate ROS include SOD, CAT, and POD (28). The decomposition of ROS depends on the antioxidant enzymes, such as CAT, and is closely related to the enhancement of the biological control ability of antagonistic yeast (29). As an important part of the antioxidant defense system, SOD can effectively remove ROS and reduce cell death (6). The POD can effectively remove excessive ROS in cells and maintain the intracellular redox state (30). As can be seen in Fig. 4, compared with the control, the activities of CAT, SOD, and POD in *P. kudriavzevii* induced by 0.15 g L^−1^ Ca ascorbate were significantly increased during the whole experiment, protecting yeast from oxidative damage. The expression levels of antioxidative enzyme-related genes were consistent with the activities of antioxidant enzymes in the present study. In the previous study, vitamin C significantly increased the antioxidant enzyme activity of *Pichia caribbica* and improved its biological control effect (6). In this study, the *CAT1*, *SOD2* and *PRXIID* gene expression levels of yeast induced by vitamin C were also measured, as well as those with the Ca ascorbate and CaCl_2_ treatments. Compared with the control, the expression of *CAT1* in *P. kudriavzevii* was significantly upregulated in all treatment groups, but the expressions of *SOD2* and *PRXIID* were significantly upregulated only in the Ca ascorbate treatment group (an increase in the CaCl_2_ and vitamin C treatments relative to the control was not significant). The above results indicate that Ca ascorbate can improve the activity of antioxidant enzymes, which may be a possible mechanism for improving the biocontrol efficacy of yeast. The enhancing effect was higher in the Ca ascorbate than the vitamin C treatment, presumably because the addition of Ca^2+^ enhanced the antioxidant capacity of Ca ascorbate.

Colonization ability is a direct factor in the biocontrol efficacy of antagonistic yeasts (31). The rapid growth of yeast on the fruit surface was conducive to the competition for nutrients and space to inhibit the growth of mold. The present study indicates that Ca ascorbate improved the population dynamics and growth rate of *P. kudriavzevii* both in the NYDB and on the cherry tomato fruit (Fig. 2 and 3), likely contributing to the enhancement of the biocontrol efficacy of *P. kudriavzevii*. The population reached the highest growth at 96 h in NYDB but at 48 h on cherry tomato, presumably because of a larger nutrient supply in NYDB. Previous studies showed that exogenous 2% (wt/vol) CaCl_2_ treatment had no significant effect on the growth of Cryptococcus laurentii either *in vitro* or *in vivo* (19) or that of Candida guilliermondii and Pichia membranifaciens *in vivo* (32). However, the 0.15 g L^−1^ CaCl_2_ treatment enhanced the colonization ability of yeast both *in vitro* and *in vivo* in our study. These results may be related to the inconsistent responses of different yeasts to CaCl_2_ and the different concentrations of CaCl_2._ A previous report indicated that vitamin C can significantly improve the colonization ability of *P. caribbica* on the surface of apples (13). In the present study, the vitamin C treatment also increased the colonization ability of yeast, but not to the same extent as Ca ascorbate. *P. kudriavzevii* multiplied rapidly in response to induction of antioxidant (Ca ascorbate) and Ca^2+^, which may be related to the increase of antioxidant enzyme activity and the regulation of physiological activities of yeast by antioxidant (Ca ascorbate) and Ca^2+^.

Fungal cell walls are essential for maintaining cell integrity. *Pga62* encodes an O-glycosylated protein located in the cell wall, contributing to the formation and stability of the cell wall (33). In our study, calcium ascorbate treatment significantly increased the expression of *Pga62* throughout the experimental period, whereas the CaCl_2_ treatment group showed the strongest upregulation at 96 h, which helped to promote chitin synthesis and maintain yeast cell wall integrity and presumably could be beneficial to cell wall synthesis, thereby increasing yeast cell survival. There have been reports that Ca^2+^ acts as a ubiquitous intracellular messenger, regulating cell proliferation, programmed death, and many other processes in eukaryotic cells (34). Following influx, Ca^2+^ binds to calmodulin, creating a complex able to activate calcineurin and therefore promote expression of specific genes required for cell proliferation and response to pheromones (35). Other studies showed that external addition of Ca^2+^ increased the cell survival rate of Debaryomyces hansenii and *Pichia* and improved the biocontrol effect of yeast (36). Vitamin C also significantly increased the expression of *Pga62* at 96 h, but not as much as Ca ascorbate and CaCl_2_. In summary, the Ca ascorbate treatment had a better induction effect on yeast colonization than vitamin C in the present study, and it is speculated that Ca^2+^ has a synergistic effect in promoting proliferation.

Energy metabolism and glucose metabolism are closely related to the colonization of yeast and the utilization of nutrients, which may improve the biocontrol efficacy of yeast (13). We measured the activity of succinate dehydrogenase to analyze the metabolic activity of *P. kudriavzevii*. Figure 6 shows that the Ca ascorbate and CaCl_2_ treatments improved the metabolism of yeast. Succinate dehydrogenase is an important enzyme in the mitochondrial respiratory system that produces ATP to provide sufficient energy for yeast cell activity by dehydrogenating succinate to fumarate and promoting yeast cell metabolism and nutrient utilization (37). Yang et al. (28) showed that vitamin C increased the metabolic activity of yeast under oxidative conditions. Similarly in the present study, the vitamin C treatment also increased the metabolic activity of cells to various degrees, but the effect was not as strong as that of Ca ascorbate.

*PET100p* is a nuclear gene specific to the assembly of cytochrome *c* oxidase, which is related to the respiratory metabolism in mitochondria of yeast cells (38). Decreased cytochrome *c* oxidase activity causes mitochondrial dysfunction, which may be accompanied by increased accumulation of ROS, accelerating the senescence and apoptosis of yeast cells (39). Figure 7C showed that the Ca ascorbate treatment induced the high expression of *PET100p*, which may reduce ROS in cells, increase energy yield, and promote the utilization of nutrients.

*HXT5* encodes xylose kinase involved in the pentose phosphate pathway, which provides energy for the growth of yeast cells (40), thereby improving the biocontrol efficacy of yeast. Figure 7A showed that the expression of *HXT5* in yeast cells was significantly upregulated after induction by Ca ascorbate and CaCl_2_, which accelerated the utilization of glucose by yeast. Alcohol dehydrogenase (ADH) is a key enzyme in ethanol metabolism, catalyzing the reversible conversion of a variety of alcohols into aldehydes and ketones (41) and increasing the utilization of hexose to provide more energy for yeast cells. The gene expression of *ADH6* was significantly upregulated in the treatments with Ca ascorbate and CaCl_2_ (Fig. 7B).

Researchers found that vitamin C can promote the metabolic ability of *Pichia caribbica*, which is related to the improvement of yeast biocontrol effectiveness (13). In our study, the expression of *PET100p* and *HXT5* was upregulated by 0.25 g L^−1^ and 0.15 g L^−1^ vitamin C at 96 h and 48 h, respectively, but the expression was lower than in the Ca ascorbate treatment group. The expression of *ADH6* was significantly induced by the vitamin C treatment throughout the treatment period, but not as effectively as with Ca ascorbate. In general, the metabolic activity of *P. kudriavzevii* was improved after the treatments with Ca ascorbate and CaCl_2_, improving the utilization efficiency of nutrients and the nutritional competition ability of yeast against the pathogen. Vitamin C also enhanced the metabolic capacity of *P. kudriavzevii*, but not as much as Ca ascorbate, which may be due to the synergistic effect of Ca^2+^.

Nutrition and space competition is one of the main mechanisms of biological control. The reproduction and metabolic capacity of yeast cells are directly related to their nutrition and space competitiveness. The 0.15 g L^−1^ Ca ascorbate treatment improved the colonization ability of yeast cells and accelerated the utilization of nutrients and thus the biocontrol efficacy of yeast.

The 0.15 g L^−1^ Ca ascorbate treatment can be a strategy to improve the biocontrol efficacy of *P. kudriavzevii* against cherry tomato gray mold. The mechanism is related to improving the ROS scavenging capacity of yeast cells and strengthening their competitiveness for nutrients by improving energy metabolism, cell wall synthesis, and glucose metabolism. Compared with the control, the effect of 0.15 g L^−1^ of Ca ascorbate on the biocontrol efficacy of yeast was more significant. In addition to the ability of antioxidant enzymes, Ca^2+^ also had a synergistic promotion effect on the biocontrol efficacy by regulating yeast activity.

## MATERIALS AND METHODS

### Fruits and treatment.

Mature red cherry tomato fruits (Lycopersicon esculentum *Mill*, Minny tomato) without mechanical damage or infection were selected according to uniformity and ripeness. The surface disinfection of fruit was done in 0.1% vol/vol sodium hypochlorite for 2 min, followed by rinsing thoroughly with tap water. Subsequent experiments were performed after air drying at room temperature.

### Yeast and pathogen.

The antagonistic yeast *P. kudriavzevii* strain Ckrus 33 08 15 05 was isolated from naturally fermented congee the in western region of Inner Mongolia. The yeast was cultured in nutrient yeast dextrose broth medium (NYDB; 8 g nutrient broth, 5 g yeast extract, 10 g glucose in 1 L distilled water) with shaking for 24 h (200 rpm, 28°C) (42). Yeast cells were then collected by centrifugation (1,000 × *g* at 4°C for 15 min), and cell suspension was adjusted to 1 × 10^8^ cells mL^−1^.

The pathogen *B. cinerea* (CGMCC 3.4584) was cultured on potato dextrose agar (PDA) at 25°C for 7 days prior to use. The culture was flooded with sterile distilled water to obtain spores, and the concentration of the suspension was adjusted to 1 × 10^4^ spores mL^−1^.

### Induction of yeast.

First, 1 mL of the *P. kudriavzevii* cell culture described above was added to 50 mL of NYDB medium containing 0 g L^−1^, 0.05 g L^−1^, 0.10 g L^−1^, 0.15 g L^−1^, 0.20 g L^−1^, 0.25 g L^−1^, 0.50 g L^−1^, or 1 g L^−1^ of Ca ascorbate, 0.15 g L^−1^ CaCl_2_, 0.15 g L^−1^ vitamin C, and 0.25 g L^−1^ vitamin C. Then the cells were harvested by centrifugation at 1,000 × *g* for 15 min (4°C) and washed twice in sterile distilled water to remove the growth medium and Ca ascorbate. The cells were resuspended in sterile distilled water, counted on a hemocytometer, and then adjusted to 1 × 10^8^ cells mL^−1^.

**Ca ascorbate treatment effects on the biocontrol efficacy of *P. kudriavzevii* against *B. cinerea* development in harvested cherry tomato.** Cherry tomato fruits were randomly divided into 27 groups of 20 (for 9 treatments in 3 replicates). The wound (5 mm diameter by 3 mm deep) was created at the equator of each cherry tomato fruit using a sterilized punch. Yeasts were induced as described above in the section on induction of yeast, and each wound was treated with 15-μL solutions as follows: (i) cell suspension of *P. kudriavzevii* (1 × 10^8^ cells mL^−1^); (ii) cell suspension of *P. kudriavzevii* (1 × 10^8^ cells mL^−1^) induced with different concentrations of Ca ascorbate at 0.05 g L^−1^, 0.10 g L^−1^, 0.15 g L^−1^, 0.20 g L^−1^, 0.25 g L^−1^, 0.50 g L^−1^, or 1 g L^−1^; and (iii) sterile distilled water as the control. After 3 h, 15 μL of the *B. cinerea* suspension (1 × 10^4^ spores mL^−1^) was added to each wound. After drying at room temperature, fruits were individually packed in plastic boxes, and stored at 25°C and 90% relative humidity (RH). The disease incidence and lesion diameter on cherry tomato was recorded 24, 48, and 72 h after inoculation. Fruits were determined to be infected when mycelium was observed on wounds (43). Disease incidence was calculated according to the method reported by Apaliya et al. (44) with some modifications. The formula was number of infected cherry tomatoes/total number of cherry tomatoes × 100%. For quantitative analysis of disease progression, the area under the disease-progress curve (AUDPC) was calculated according to Jeger et al. (45) as

$$\text{AUDPC = }\;\Sigma_{i\;=\;1}^{n\;-\;1}\;[({\text{Y}}_{i}\;+\;{\text{Y}}_{i+1})/2][{\text{X}}_{i+1}\;-\;{\text{X}}_{i}]$$

where *n* is the number of evaluations, Y*_i_* is the disease incidence, and X*_i_* is the number of hours after infection at each evaluation. The experiment was conducted three times with three replicates each time, and each replicate comprised 20 cherry tomato fruits.

### Population dynamics and cell growth rate of *P. kudriavzevii*.

**(i) *In vitro* test.** The growth of *P. kudriavzevii* was tested following the reported method (46) with some modifications. An aliquot (1 mL) of the cell suspension of *P. kudriavzevii* (1 × 10^8^ cells mL^−1^) was added to NYDB combined with 0.15 g L^−1^ CaCl_2_ (induced in the same way as in the case of Ca ascorbate), 0.15 g L^−1^ Ca ascorbate, and no addition (as the control). After 24 h, 1 mL of yeast cell suspension (1 × 10^8^ cells mL^−1^) was added to NYDB at 28°C on a shaker (200 rpm). Cells were collected after 24, 48, 72, and 96 h. The density of yeast cells was expressed as the absorbance at 600 nm. Experiments were replicated three times with three samples per replicate.

**(ii) *In vivo* test.** The growth of *P. kudriavzevii* on cherry tomatoes was tested following the reported method (13) with some modifications. The wound (5 mm diameter by 3 mm deep) was created at the equator of each cherry tomato fruit using a sterilized punch. Yeasts were induced as described above in the section on induction of yeast, and each wound was treated with 15-μL solutions as follows: (i) cell suspensions of *P. kudriavzevii* (1 × 10^8^ cells mL^−1^) harvested from NYDB supplemented with 0.15 g L^−1^ Ca ascorbate, 0.15 g L^−1^ CaCl_2_, 0.15 g L^−1^ vitamin C, or no addition and (ii) sterile distilled water as the control. After drying at room temperature for 2 h, fruits were individually packed in plastic boxes; samples were taken at 24, 48, 72, and 96 h.

Yeast counting was done using the plate counting method. A sterile cutter with a diameter of 0.5 cm was used to sample the fruit wound and put it into an appropriate amount of sterile water for grinding, mixing, and gradient dilution. A 10-μL dilution was smeared on an NYDA plate and cultured at 28°C for 24 h before counting. The experiment was repeated three times with three samples per replicate.

### Determination of antioxidant enzyme activity of *P. kudriavzevii*.

Yeast was prepared as described above and cultured in the NYDB medium or the NYDB supplemented with 0.15 g L^−1^ Ca ascorbate. After 24 h, 1 mL of yeast cell suspension (1 × 10^8^ cells mL^−1^) was added to NYDB medium at 28°C on a shaker (200 rpm). Yeast cells were collected after 24, 48, 72, and 96 h, centrifuged at 10,000 × *g* for 10 min, washed with sterile distilled water 3 times, and adjusted to 1 × 10^8^ cells mL^−1^.

The same amount of yeast was taken and centrifuged to remove the supernatant, ground in liquid nitrogen, and suspended in chilled phosphate-buffered saline (PBS) (50 mM, pH 7.8, containing 5 mM dl-dithiothreitol and 5% polyvinylpolypyrrolidone). The cell suspension was centrifuged at 10, 000 × *g* for 20 min (4°C); the supernatant was used for enzyme assays.

The activities of CAT and SOD were determined using assay kits (Solarbio, Beijing, China) according to the manufacturer’s instructions. The POD activity was measured according to the method of Mahunu et al. (47), and 1 unit was defined as an increase in 470 nm absorption of 0.01 per minute. Assays were done in three independent replicates.

The activities of CAT, SOD, and POD were expressed as units per milligram of protein. Protein content was measured as described by Bradford (48), using bovine serum albumin (BSA) as the standard.

### Metabolic activity analysis of *P. kudriavzevii*.

The metabolic activity of *P. kudriavzevii* was determined according to the method of Camejo et al. (49). Yeasts were treated as described above in the section on induction of yeast (yeasts were induced by 0.15 g L^−1^ Ca ascorbate, 0.15 g L^−1^ CaCl_2_, 0.15 g L^−1^ vitamin C, 0.25 g L^−1^ vitamin C, or no addition); the same amount of yeast was resuspended in 1 mL of PBS (0.05 M, pH 7.5), supplemented with 350 μL 2,3,5-triphenyl-tetrazolium chloride, vortexed, and stored in the dark for 20 h. After centrifugation at 10,000 × *g* for 10 min, samples were washed twice with sterile distilled water. An equal volume of glass beads and ethanol-acetone 500 μL (1:1) was vortexed repeatedly four times to disrupt the cells. The samples were extracted with acetone twice, and the absorbance was measured at 425 nm (the range of 425 to 600 nm was scanned first to find an optimum). The results were analyzed from three independent replicates.

### Transcriptional analysis of yeast genes.

**(i) Treatments.** The treatment and collected methods of yeast cells were similar to that described above (yeasts were induced by 0.15 g L^−1^ Ca ascorbate, 0.15 g L^−1^ CaCl_2_, 0.15 g L^−1^ vitamin C, 0.25 g L^−1^ vitamin C, or no addition) and sampled at 48 and 96 h. For RNA extraction and reverse transcription, the hot phenol method was used to extract RNA from *P. kudriavzevii* (50) with some modifications. A Colibri microvolume spectrometer was used to measure the concentration and quality (Titertek-Berthold, Pforzheim, Germany). The cDNA was synthesized using a PrimeScript real-time (RT) reagent kit with genomic DNA (gDNA) Eraser (RR047A; TaKaRa, Dalian, China) according to the manufacturer’s protocol.

**(ii) RT-qPCR.** The reverse transcriptase quantitative PCR (RT-qPCR) was performed to evaluate the relative expression levels of antioxidant enzyme genes (*CAT1*, *SOD2*, and *PRXIID*), cell proliferation-related genes (*PET100p* and *Pga62*), and cellular energy metabolism-related genes (*HXT5* and *ADH6*). The β-actin gene of *P. kudriavzevii* was used as the internal reference. The gene-specific primers in our study are listed in Table S1 in the supplemental material. The RT-qPCR was carried out using a kit (SYBR Premix *Ex Taq* [TliRNaseH Plus]) in a final volume of 20 μL on a Bio-Rad CFX96 Touch real-time PCR detection system (Bio-Rad, Singapore). The PCR procedure comprised step 1 at 95°C for 30 s and step 2 at 95°C for 5 s and 60°C for 34 s, repeated 40 times. The melting curve was done at 95°C for 15 s, at 60°C for 60 s and at 95°C for 15 s. The gene expression level was calculated by the 2^–ΔΔ^*^CT^* method (51). The transcript abundance of genes of different induced groups was expressed as fold changes in relation to the control at each time point. Each sample is composed of three biological replicates.

### Statistical analyses.

All the experiments were repeated three times, and three replicates were conducted for each assay. The data shown here were obtained in a single experiment but were representative of three independent experiments yielding similar results. All results were analyzed by analysis of variance (ANOVA) using the statistical program SPSS/PC version II.x, (SPSS, Inc., Chicago, IL, USA), and Duncan’s multiple-range test was used for mean separation. The statistical significance was assessed at *P < *0.05.
