# Supplementary material for: Enhancement of Biocontrol Efficacy of Pichia kudriavzevii Induced by Ca Ascorbate against Botrytis cinerea in Cherry Tomato Fruit and the Possible Mechanisms of Action
Source: Microbiol Spectr. 2021 Dec 22;9(3):e01507-21. doi: 10.1128/spectrum.01507-21 (PMC8694134; doi:10.1128/spectrum.01507-21)
Supplement: SUPPLEMENTAL FILE 1 — Supplemental material. Download SPECTRUM01507-21_Supp_1_seq9.pdf, PDF file, 0.7 MB [file spectrum01507-21_supp_1_seq9.pdf]

TABLE 1 Gene-specific primer sequences used for real-time quantitative PCR

Gene Name	Accession number	Forword primer(5'-3')	Reverse primer(5'-3')
β-actin	BOH78_166	GTACCACCATGTTCCCAGGT	TGGAAGGTGGATAGAGATGC
CAT1	BOH78_4835	TTGGGCGCAACTCCTAACTA	CGGCCTAAAACGTCCCAAAA
SOD2	BOH78_1681	ATTGCGCCATTGTCGGTGTTCT	GCGGTGGTAAGTTGCCATCTGA
PRXIID	BOH78_4088	ACCCGTGCCTGGAGTGAAGATT	CTTGAGCCACGAATGCCGTCTT
HXT5	MG548729	CGGATCTCTTGGTTCTCGCA	GCAAGATGGAAACGACGCTC
ADH6	BOH78_5134	GCCGAGGTCACAGCAATATCCA	CGTGTCCATGTGCTCCTCCATT
PET100p	XM_033909437	TGGAGCCTCGGAAGCTGGAAC	TACACCACCTACTGCCCACTTACC
Pga62	XM_715422	TGGAGCCTCGGAAGCTGGAAC	TACACCACCTACTGCCCACTTACC
